# Bioactive glasses as bone substitutes for animal critical defects: A systematic review

**DOI:** 10.1590/0103-644020256464

**Published:** 2025-10-24

**Authors:** Luiza Bastos Nozari, Hiskell Francine Fernandes e Oliveira, Gabriela Dandaro Marinho, Joel Ferreira Santiago, Márcia Martins Marques, Maria Cristina Zindel Deboni, Emanuela Prado Ferraz

**Affiliations:** 1 Department of Prothesis, Oral and Maxillofacial Surgery and Traumatology. School of Dentistry, University of Sao Paulo, Sao Paulo, Brazil; 2 Department of Oral and Maxillofacial Surgery and Periodontology, School of Dentistry of Ribeirao Preto, University of Sao Paulo, Sao Paulo, Brazil; 3 Department of Dental Materials and Prosthesis, School of Dentistry of Ribeirao Preto, University of Sao Paulo, Sao Paulo, Brazil; 4 Aachen Dental Laser Center, Sigmund Freud University, Wien, Austria

**Keywords:** Bioactive glass-based, bone substitute, regeneration, uCT

## Abstract

Bioactive glasses (BGs) have garnered attention for their ability to bond with native bone tissue and exhibit osteoconductive properties. Despite studies investigating BGs for bone repair, results have been inconsistent, and an ideal formulation for regenerating critical defects has not been established. This systematic review aimed to analyze evidence regarding BG-based biomaterials for bone regeneration in animals with critical defects. The review followed PRISMA guidelines and was registered in PROSPERO (CRD42022325250). A literature search was conducted using PubMed, Web of Science, and Scopus, based on inclusion and exclusion criteria. The risk of bias was assessed using the SYRCLE RoB Tool for Animal Studies. Nine of the 1,467 identified studies were included. Most studies have indicated that bone defects created in rats and rabbits, and treated with BG-based biomaterials, show improved bone formation compared to untreated defects. Key parameters included bone volume, percentage of bone volume, bone mineral density, trabecular number, thickness, and separation. Although most studies indicate that BG-based biomaterials enhance bone formation in critical-sized defects compared to empty controls, study heterogeneity prevents the drawing of definitive conclusions. This review emphasizes the importance of standardized assessment methods to produce reliable research reports that provide robust evidence, ensuring accurate efficacy assessments and ultimately leading to more effective clinical interventions.

## Resumo

Vidros bioativos (BVs) ganharam atenção por sua capacidade de se ligar ao tecido ósseo nativo e suas propriedades osteocondutoras. Apesar de estudos investigando BVs para reparo ósseo, os resultados têm sido inconsistentes, e uma formulação ideal para regenerar defeitos críticos não foi estabelecida. Esta revisão sistemática teve como objetivo analisar evidências sobre biomateriais baseados em BG para regeneração óssea em animais com defeitos críticos. A revisão seguiu as diretrizes PRISMA e foi registrada no PROSPERO (CRD42022325250). Uma busca bibliográfica foi conduzida usando PubMed, Web of Science e Scopus, com base em critérios de inclusão e exclusão. O risco de viés foi avaliado usando a ferramenta SYRCLE RoB para estudos em animais. Nove dos 1.467 estudos identificados foram incluídos. A maioria dos estudos indicou que defeitos ósseos confeccionados em ratos e coelhos e tratados com biomateriais baseados em BG, tiveram aumento da formação óssea em comparação aos defeitos não tratados. Os principais parâmetros incluíram volume ósseo, porcentagem de volume ósseo, densidade mineral óssea, número trabecular, espessura e separação. Embora a maioria dos estudos indique que biomateriais à base de BV aumentam a formação óssea em defeitos de tamanho crítico em comparação com controles vazios, a heterogeneidade dos estudos impede conclusões definitivas. Esta revisão destaca a necessidade de métodos de avaliação padronizados para obter relatórios de pesquisa confiáveis que contribuam com evidências robustas, garantindo avaliações de eficácia precisas e, em última análise, resultando em intervenções clínicas mais eficazes.

**Figure:**
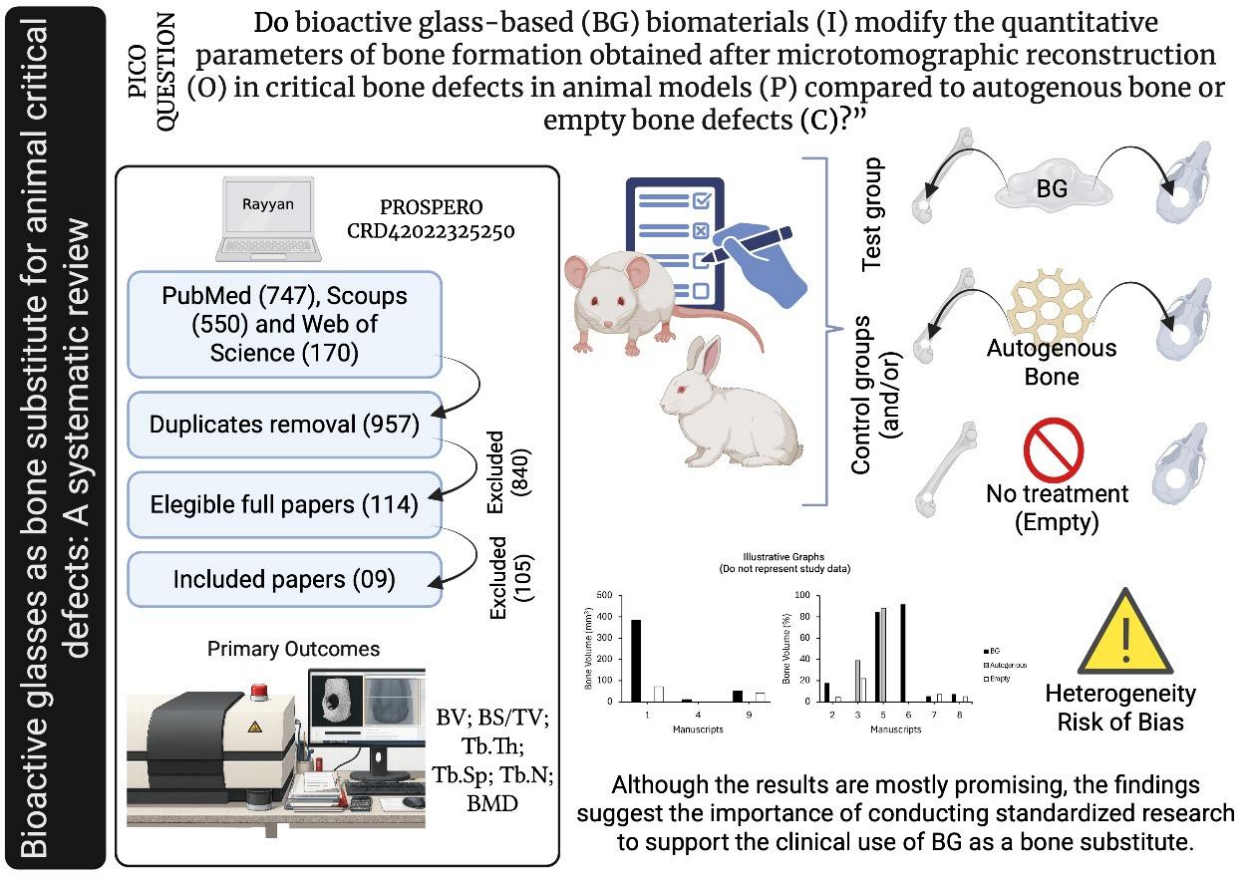

## Introduction

An optimal bone substitute should possess several key properties, such as biocompatibility and improved osteoconduction, osteoinduction, and/or osteogenesis, in addition to resorption and biodegradability, while temporarily withstanding mechanical loads ^1^ ^,^ ^2^. Each bone substitute option, including autogenous bone, allogeneic bone, and alloplastic materials, comes with its own set of advantages and disadvantages ^1^ ^,^ ^2^. Despite significant progress in the development of biomaterials that promote bone regeneration and restore aesthetics and functions, an ideal biomaterial that fulfills all requirements is currently unavailable ^2^.

Although autogenous bone is regarded as the gold standard, its main disadvantages include the risk of complications and unpredictable resorption ^1^ ^,^ ^3^. Alternatively, synthetic bone substitutes have been used to reduce such morbidity; however, their lack of osteogenic potential and high costs have limited their use ^4^. In the late '70s, a glass-based biomaterial capable of bonding to native bone tissue without a connective tissue interface was developed and classified as a bioactive material, which was patented as Bioglass 45S5^® (^ ^5^ ^,^ ^6^. Its dissolution products stimulate osteoprogenitor cells at the bone-material interface, resulting in osteoconductive and osteoinductive properties. Despite their advantages in terms of bioactivity, Hench ^(^ ^7^ found that bioactive glasses (BGs) are fragile and have sharp surfaces with limited mechanical properties. Therefore, their use for this purpose is limited to areas with low mechanical stress. Modifications to the sintering process and production of the scaffolds have been proposed; however, this reduces bioactivity. In this context, chemical elements have been incorporated into BGs to enhance their biocompatibility, osteoblast differentiation, and bone formation ^7^ ^,^ ^8^ ^,^ ^9^ ^,^ ^10^.

In the clinic, orthopedic and maxillofacial surgeons aim to find biomaterials with the above-listed properties to regenerate high-quality bone tissue in critical defects, that is, those that cannot be repaired without treatment, and to restore the patient's function and aesthetics. A thorough review of the existing literature and a careful evaluation of outcomes are required to justify their treatment choices ^1^. Modification of BG formulations over time has improved their properties, potentially making them ideal bone substitutes ^11^. However, the literature on BG-based materials consists of inconsistent reports and data, with various formulations produced using different methods ^6^ ^,^ ^9^ ^,^ ^10^.

An evaluation of treatment efficacy requires a comparison of the amount and quality of bone formed in a critical defect model with that of the gold standard or control. Another key point is that it is essential to examine how the results were obtained critically, and in recent years, high-resolution technologies have provided more accurate and reliable results ^11^ ^,^ ^12^. If no data supports this clinical demand, new investments in materials will be necessary. Based on these findings, we aimed to systematically review the scientific literature focusing on bone formation morphometric parameters from microtomographic reconstructions of critical defects created in animals and treated with BGs, comparing these outcomes with those achieved using autogenous bone.

## Material and methods

This systematic review was designed according to the PRISMA guidelines (Preferred Reporting Items for Systematic Reviews and Meta-Analyses). It was registered in the International Prospective Register of Systematic Reviews (PROSPERO) under the protocol ID CRD42022325250 ^13^. The focus of the PICO question was: “Do bioactive glass-based biomaterials (I) modify the quantitative parameters of bone formation obtained after microtomographic reconstruction (O) in critical bone defects in animal models (P) compared to autogenous bone or empty bone defects (C)?”

### Search strategy

A systematic electronic search of articles from 2009 to 2024 was conducted using PubMed, Web of Science, and Scopus, employing a Boolean search with descriptors defined according to the available databases at the Virtual Health Library (https://decs.bvsalud.org) (Box1). Studies published in English that met the criteria and filters applied were included: interventional studies; those using glass-based bioactive biomaterials alone or doped with inorganic ions; those including three-dimensional quantitative evaluation from microtomographic reconstructions of bone formation of surgically constructed critical defects; conducted on healthy mammals of any sex; and studies with a control group (defects treated with autogenous or untreated-empty defects). Exclusion criteria were: *in vitro* studies; studies conducted on humans or non-mammalian animals; those on animals with metabolic diseases; literature reviews, systematic reviews, series, and case reports; interventions in which the base materials were covered with glass and other non-ionic biomaterials; studies on anatomical defects and/or non-critical defects; studies without a control group; and studies that evaluated only qualitative or two-dimensional images of the repair.

**Box1.**
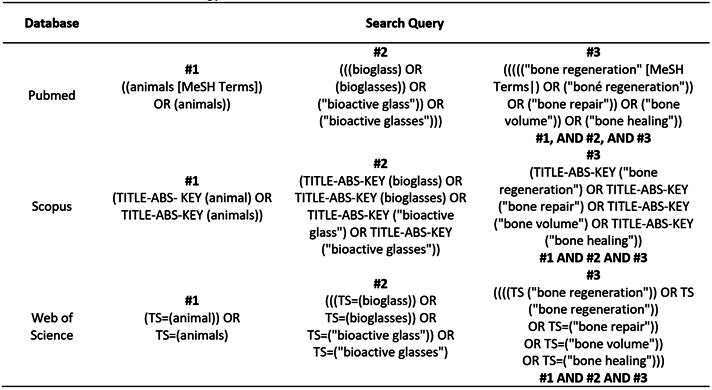
Electronic search strategy for each online database

### Study selection

Two independent reviewers (LBN and EPF) selected studies by their titles and abstracts using the web application Rayyan ^14^. Manuscripts with insufficient abstract information were considered in the next step. The same reviewers read the selected manuscripts to ensure that they met the inclusion criteria. A third reviewer (MCZD) was consulted to resolve disagreements.

### Data extraction

The data extracted were categorized into the following items: The title of the study; the study aim; population characteristics including species, sex, age, weight, type, and location of the bone defect, and sample size and number of specimens in the sample; intervention: material and associations used; and control group: autogenous and/or empty defect; Primary outcomes of bone formation were assessed according to guidelines for assessment of bone microstructure in rodents ^15^ as follows: bone volume (BV), bone density (BS/TV), trabecular thickness (Tb.Th), number of trabeculae (Tb.N), trabeculae separation (Tb.Sp), and bone mineral density (BS/TV); Secondary outcomes include histometric and angiogenesis markers as well as inflammatory and cytotoxicity markers. When data were presented in graphs without numerical descriptions, the corresponding authors were contacted. In the absence of responses, a computer program (https://plotdigitizer.com, version 4.1) was used to extract means and corresponding standard deviations from the static graphic images presented in the results sections.

### Risk of bias

The risk of bias assessment was conducted using the SYRCLE RoB Tool for Animal Studies (Systematic Review Center for Laboratory Animal Experiments) ^16^ ^,^ ^17^. Briefly, six domains were evaluated for biases, including selection, performance, detection, attrition, reporting, and other sources of bias, which were classified as “low,” “high,” or “uncertain.” When all guiding questions for a domain were “Yes” or “Probably Yes,” the study was ranked low. When answers to the domains were unclear, the study was classified as uncertain. When one of the items had negative responses, the bias for a particular domain or subdivision was considered high.

## Results

### Study selection

**Figure 1:**
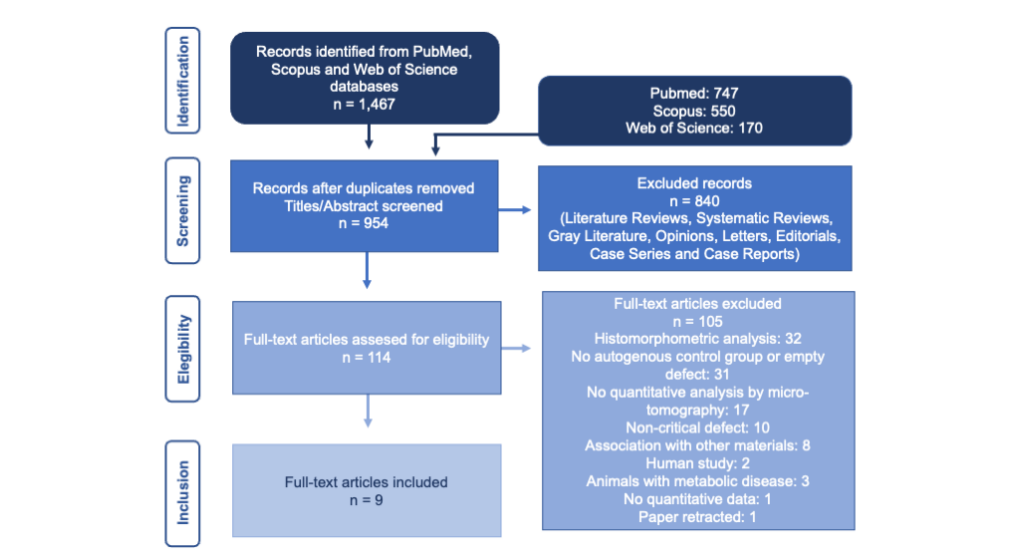
Flowchart of the search strategy and selection of articles based on exclusion criteria.

The search was initiated in May 2022 and updated in July 2024. Nine papers were deemed to meet the inclusion criteria and included in the review. A flowchart outlining the search process, study selection, and the main reasons for excluding the manuscript is shown in Figure 1.

### Included studies and their methodological analysis

The detailed aims, populations, and interventions are summarized in Box2. They were conducted in universities and research institutions in China, Brazil, Finland, and the United Kingdom, and were published between 2015 and 2021 in journals focusing on the development of biomaterials. Most studies assessed aspects such as angiogenesis, osteogenesis, or bone repair, while one study additionally investigated immunomodulation.

Among them, six studies investigated bone repair in rats ^18^ ^,^ ^19^ ^,^ ^20^ ^,^ ^21^ ^,^ ^22^ ^,^ ^23^ and three in New Zealand rabbits ^24^ ^,^ ^25^ ^,^ ^26^, with ages ranging from 6 to 24 weeks (200-500 g for rats and 2.1-3.5 kg for rabbits). The number of animals included in the studies ranged from 4 to 150, with an average of 7.11 ± 3.91 animals per group. Bone defects were categorized as critical in all studies and were predominantly created in the calvaria, followed by the femur and radius. The mean diameters of the defects were 6.6 ± 4.1 mm for calvaria, 4.2 ± 3.1 mm for femurs, and 15 mm for the radius.

These studies utilized commercially available BGs or glasses that were modified by incorporating ionic compounds and/or produced using different sintering methods. Six studies incorporated specific ions into the material: Nb ^19^ ^,^ ^20^, Cu ^24^, Sr ^18^, and Zn ^22^. Most studies used bone formation in empty defects (i.e., defects filled with clot) as the control group. However, one study used empty defects and autogenous bone as the negative and positive controls, respectively ^24^. Only one study used bone formed in defects filled with autogenous bone for comparison ^19^.

**Box2. Descriptive:**
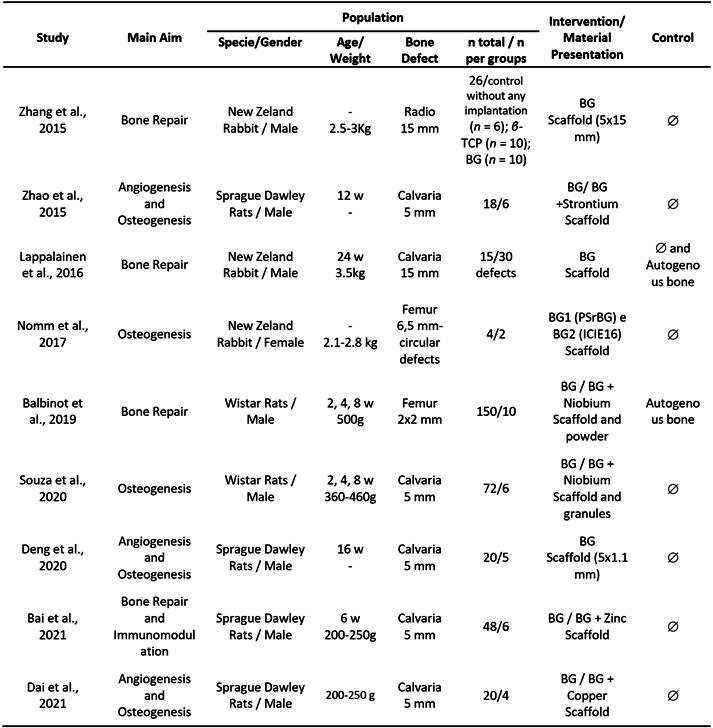
aim, population, and intervention of the included studies.

w: weeks; g: gram; Kg: kilogram; mm: millimeters; BG: Bioglass.

### Primary outcomes

The studies presented data from the morphometric analysis of bone formation after microtomographic reconstruction, as outlined in Box3. The periods selected for euthanizing the animals and analyzing bone formation varied across studies and models and ranged from 2 to 12 weeks. Most studies used a single experimental time for evaluation, while two studies assessed bone formation at two different periods, 7 and 12 weeks ^26^, and 4 and 8 weeks ^23^. Only one study examined bone formation at three distinct time points (2, 4, and 8 weeks) ^19^. The parameters described in the studies’ analysis included bone volume (mm^3^), percentage of bone volume (%), bone mineral density (BMD, mg/cm^3^), number of trabeculae (n), trabecular thickness (mm), and trabecular separation (mm)

**Box3.**
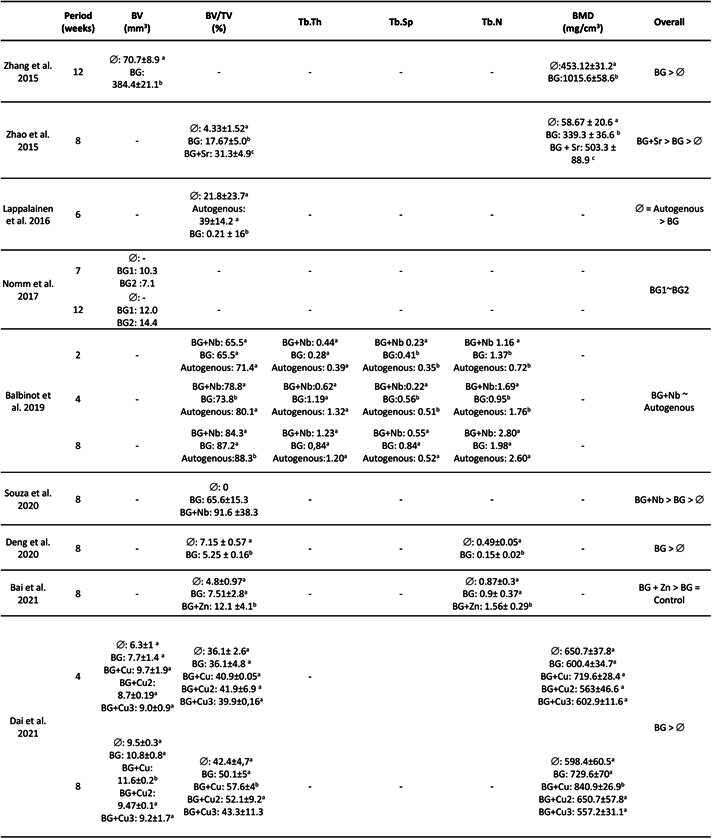
Descriptive morphometric parameters of the included studies.

BG: bioglass. (: empty defect; Cu: Cooper; Sr: Strontium; Ni: Niobium. ~: similar

Data wererounded to 01 decimal point. Different letters indicate statistical difference among groups.

The collected results, based on the parameters assessed in each study and experimental time, indicated that BG-based biomaterials (with or without ionic compounds) demonstrated superior outcomes in terms of bone formation compared to empty defects in six studies. One study showed numerically lower data ^24^, and another study reported results that were lower than those of the empty defect ^26^. These studies only provided data from the intervention groups and concluded that satisfactory bone regeneration had occurred. The only study that used autogenous bone as the control revealed comparable bone formation with BG supplemented with Nb, suggesting that the quality of the regenerated bone was preserved ^19^.

### Secondary outcomes

In addition to the primary outcomes, complementary analyses were performed in six of the nine included studies. These included heterogeneous data on immunohistochemical markers, angiogenesis, inflammatory processes, and cytotoxicity, all of which are presented in Supplementary Box S1. In general, the analyses confirmed the primary outcomes, demonstrating that bioactive glass-based biomaterials improved bone formation compared to empty defects ^21^ ^,^ ^23^ ^,^ ^25^. Additionally, these materials did not exhibit significant inflammatory factors ^21^ and showed good tissue biocompatibility, causing no damage to the evaluated tissues and organs ^20^.

### Risk of bias

**Figure 2.**
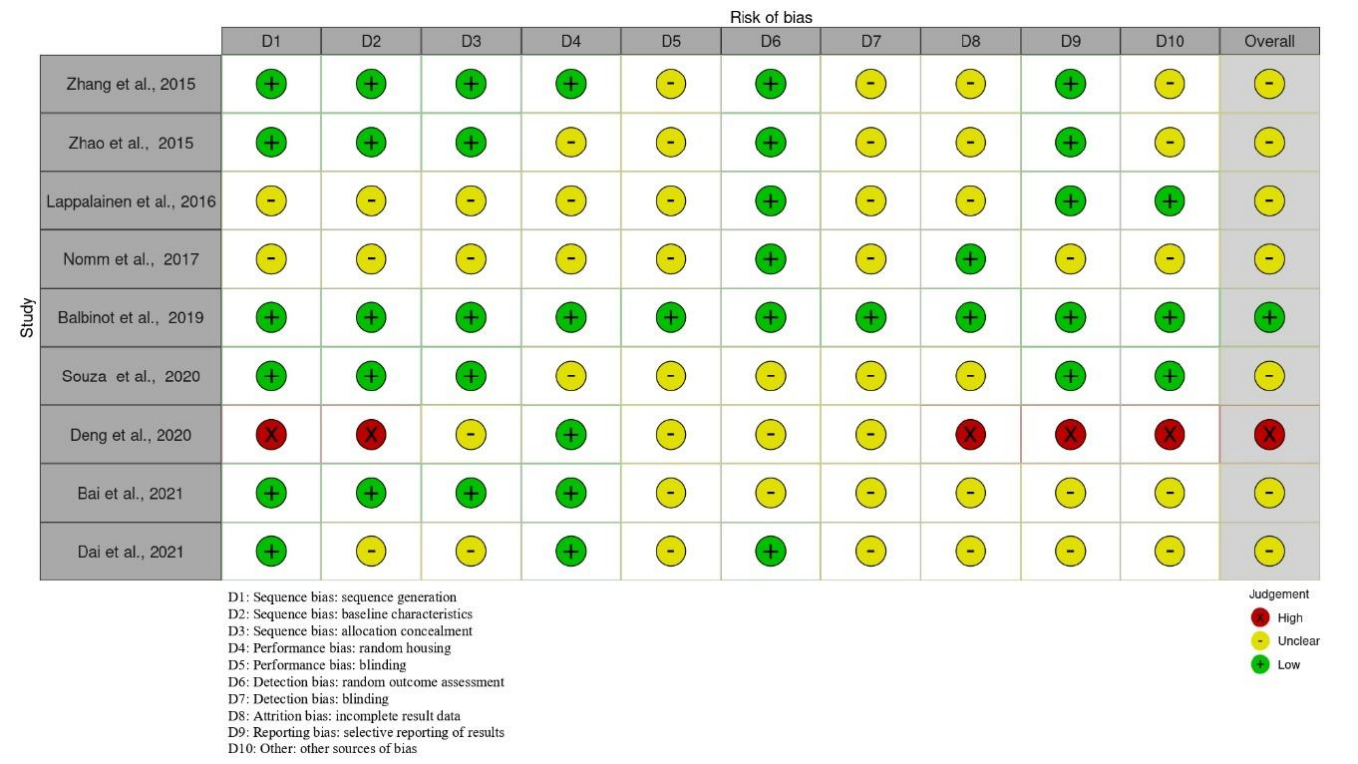
Riskof bias of the included studies, according to the assessed domains. Chart generated using the RobVis tool.

Among the included studies, Balbinot et al. ^19^ addressed the questions from each domain and were classified as having a "low risk" of bias. Most studies were classified as having “some concerns,” as they did not explicitly provide data to answer all the questions proposed by the SYRCLE guidelines ^16^. One study was classified as “high risk” due to deficiencies in various domains, including selection, attrition, reporting, and other potential sources of bias ^21^. A graph generated from responses to the SYRCLE questionnaire using the RobVis tool is shown in Figure 2, which illustrates the risk assessment for each study.

## Discussion

This study presents a systematic review of bone formation morphometric parameters obtained after microtomographic reconstructions of critical defects created in animals treated with BGs. Despite the initial inclusion of only nine out of hundreds of studies, the potential of bioactive glass-based biomaterials for bone regeneration is promising. The collective results indicate that BG-based biomaterials increased bone formation compared to empty defects filled only with clots. Of these, only two studies used autogenous bone for comparison, but they yielded conflicting outcomes ^19^ ^,^ ^24^. Although autogenous bones are considered the gold standard for bone regeneration, most studies used empty defects as controls. In one of the included articles, the descriptions of the groups needed to be clearer, which could lead to misinterpretation of the results compared with the control group ^21^, highlighting the importance of clarity in experimental designs and reporting to ensure accurate interpretation of study results.

Until the mid-1990s, qualitative and quantitative evaluations of bone tissue were conducted using 2D methods and/or radiographic evaluations ^11^. With the advent of high- and ultra-high-resolution computed tomography scanners and software, three-dimensional evaluations have enabled new quantitative and qualitative analyses, providing valuable insights into bones ^12^. Studies that use histometric data should not be invalidated; due to the need for precise and standardized data collection, this review included only articles published from 2009 onward, when the guidelines were introduced ^15^. Studies presenting only histometric data were not included. The increase in popularity and extensive adoption of 3D analysis systems has led to the necessity for standardization and consistency in variables describing for comparison between studies ^12^. Despite this trend, none of the included studies presented the recommended morphometric parameters to assess bone formation ^(^ ^15^, and less than half of the studies presented numerical data. Furthermore, not all studies described the equipment's resolution and voltage, the slices' thickness, the volume and region of interest, or the software used to perform the analyses. The lack of comprehensive reporting may affect the ability to make direct comparisons and draw definitive conclusions across studies, highlighting the importance of adhering to established guidelines and providing comprehensive data for future research.

The included articles were predominantly published in high-impact journals in biomaterials, focusing on the development and characterization of materials with medical and dental applications, as well as their initial biological responses. The primary focus was on understanding how biomaterials perform and how they can be applied to bone regeneration and repair, rather than directly comparing them with established gold-standard treatments. The rate and pattern of bone formation, a key aspect of our research, can vary significantly due to local and systemic factors, including bone type, defect size, age, sex, and species ^27^ ^,^ ^28^. Defects in the calvaria of small rodents are suitable models for evaluating biomaterials in particulate form. In contrast, defects in long bones are indicated in the assessment of materials in a 3D format that must withstand mechanical loads ^28^. The significant heterogeneity in animal models and analysis periods of the studies included in this review makes comparing the variables between studies a challenging task. It may contribute to variations in the observed outcomes. Additionally, the calculation of sample power, a crucial methodological step in study planning, was not included in some studies, which may explain the absence of statistical differences among groups ^29^.

Various formulations of bioactive glass-based materials have been studied and used to improve their physicochemical and biological properties ^10^ ^,^ ^30^. This review focused solely on BGs, without association with other materials. However, studies exploring the incorporation of ions (Nb, Cu, Mo, and Sr) into quaternary compounds for BG formulation, without the association of other biomaterials, were included. Most studies have demonstrated that the incorporation of ions leads to improved bone formation compared with the base material alone and/or the empty defect, indicating the potential of incorporating ions to enhance the performance of BG-based biomaterials for bone regeneration, making them promising candidates for further research and clinical applications ^20^ ^,^ ^30^. However, a meta-analysis of the data to corroborate this statement could not be conducted.

Although they did not provide relevant additional information, the secondary outcomes confirmed that BG-based biomaterials outperformed empty defects in promoting bone formation, with limited inflammatory response and tissue biocompatibility, supporting their safety and efficacy for bone regeneration. However, comparative analyses were unfeasible due to the variability in data, methods, and biases in the included studies could have been more feasible. Due to the considerable heterogeneity among the included studies, including variations in animal models, defect sizes, material formulations, evaluation methods, and outcome measures, a meta-analysis was deemed unfeasible, as these differences precluded meaningful data integration and quantitative synthesis.

Minimizing biases in research design and reporting is crucial for the accuracy and reliability of the outcomes, especially in animal studies. Not all bias risk domains affect results; studies using animals, blinding the sample, and operators during group allocation may be less critical. One study explicitly stated this, resulting in a low-risk assessment ^19^. However, blinding the evaluator collecting data is crucial to reduce detection bias, as failure can increase the odds ratio by about 36% ^31^. Attrition bias, such as unreported animal losses, generally posed minor concerns in this review, with only two studies providing clear information and thus assessed as low risk ^19^ ^,^ ^26^. In contrast, one study did not specify the number of animals in the control group, raising concerns about attrition bias. It also exhibited reporting bias by not presenting all relevant findings and labeling them high-risk ^21^. Similarly, reporting bias was observed in this study, as it claimed to evaluate outcomes at the 12-week mark but failed to present these results, only providing data for up to 8 weeks.

One limitation of this review is that it only includes publications in English, which may introduce language bias. Additionally, significant variations in methodology, particularly regarding animal models, defect sizes, biomaterial formulations, and outcome measures, prevented data pooling and the conduct of a meta-analysis. To improve comparability and enhance the overall quality of evidence, future pre-clinical studies should adopt standardized protocols. Developing new treatments is a complex process that requires evaluations of treatment effectiveness, making it essential to utilize standard reference data ^32^. By adhering to standardized reporting guidelines and incorporating comprehensive data, researchers can contribute to the accumulation of robust evidence, ensuring the reliability and credibility of their findings. This collective effort will advance our understanding of bone regeneration treatments and lead to more effective clinical interventions for patients.

## Conclusion

Most studies have demonstrated that BG-based biomaterials improved the quantitative parameters of bone formation in critical-sized defects compared to untreated controls. Nevertheless, the significant heterogeneity among studies prevents definitive conclusions regarding the efficacy of BG-based materials for regenerating critical-sized defects in animals. While this systematic review provides valuable findings, it highlights the need for standardized and high-quality research to support the clinical application of BG as a bone substitute.

## Figures and Tables

**Box S1:**
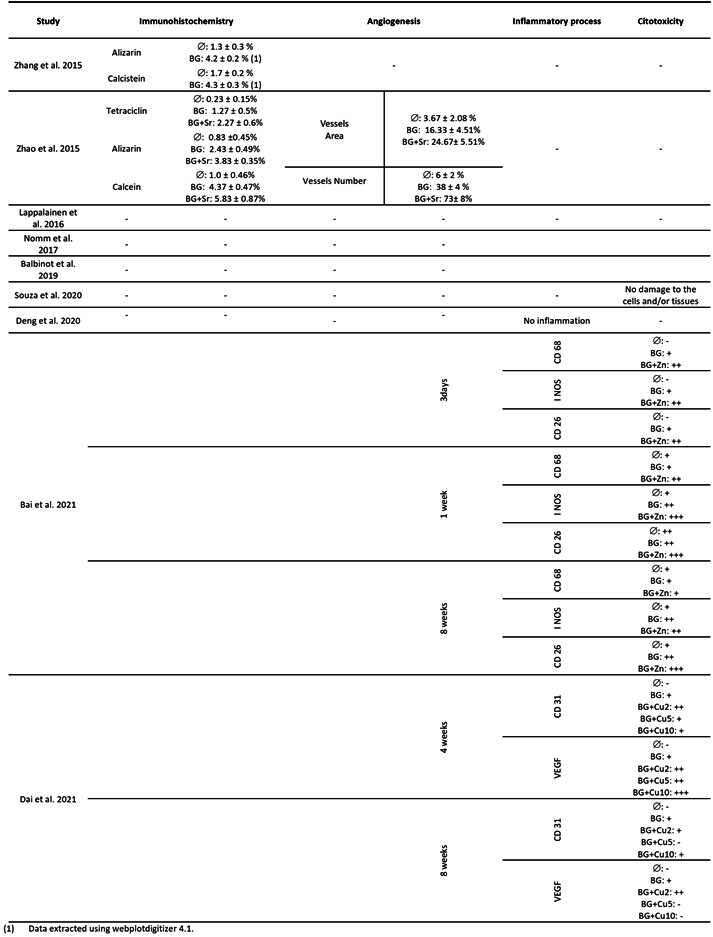
Secondary outcomes of the included studies.

## References

[1] Sanz M, Dahlin C, Apatzidou D, Artzi Z, Bozic D, Calciolari E (2019). Biomaterials and regenerative technologies used in bone regeneration in the craniomaxillofacial region: Consensus report of group 2 of the 15th European Workshop on Periodontology on Bone Regeneration. J Clin Periodontol.

[2] Tang G, Liu Z, Liu Y, Yu J, Wang X, Tan Z (2021). Recent Trends in the Development of Bone Regenerative Biomaterials. Front. Cell Dev Biol.

[3] Miron RJ (2000). Optimized bone grafting. Periodontol.

[4] Fernandez de Grado G, Keller L, Idoux-Gillet Y, Wagner ,Q, Musset AM, Benkirane-Jessel N (2018). Bone substitutes: a review of their characteristics; clinical use; and perspectives for large bone defects management. J Tissue Eng.

[5] Hench LL SR, Splinter RJ, Allen WC, Greenlee TK (1971). Bonding mechanisms at the interface of ceramic prosthetic materials. J Biomed Mater Res.

[6] Li L, Hu H, Zhu Y, Zhu M, Liu Z (2019). 3D-printed ternary SiO2CaOP2O5 bioglass-ceramic scaffolds with tunable compositions and properties for bone regeneration. Ceramics International.

[7] Hench LL (2006). The story of bioglass. J Mater Sci Mater Med.

[8] Lizzi F, Villat C, Attik N, Jackson P, Grosgogeat B, Goutaudier C (2017). Mechanical characteristic and biological behaviour of implanted and restorative bioglasses used in medicine and dentistry: A systematic review. Dent Mater.

[9] Lehman LFC, de Noronha MS, Diniz IMA, Costa da, Silva RMF, Andrade AL, de Sousa Lima LF (2019). Bioactive glass containing 90% SiO2 in hard tissue engineering: An in vitro and in vivo characterization study. Tissue Eng Reg Med.

[10] Fiume E, Barberi J, Verné E, Baino F (2018). Bioactive glasses: From parent 45S5 composition to scaffold-assisted tissue-healing therapies. J Funct Biomater.

[11] Chappard D, Retailleau-Gaborit N, Legrand E, Baslé MF, Audran M (2005). Comparison insight bone measurements by histomorphometry and microCT. J Bone Miner Res.

[12] Akhter MP, Recker RR (2021). Bone.

[13] Page MJ, McKenzie JE, Bossuyt PM, Boutron I, Hoffmann TC, Mulrow CD (2021). The PRISMA 2020 statement: an updated guideline for reporting systematic reviews. BMJ.

[14] Ouzzani M, Hammady H, Fedorowicz Z, Elmagarmid A (2016). Rayyan-a web and mobile app for systematic reviews. Syst Rev.

[15] Bouxsein ML, Boyd SK, Christiansen BA, Guldberg ,RE, Jepsen KJ, Müller R (2010). Guidelines for assessment of bone microstructure in rodents using micro-computed tomography. J Bone Miner Res.

[16] Hooijmans CR, Rovers MM, de Vries RB, Leenaars M, Ritskes-Hoitinga M, Langendam MW. (2014). SYRCLE's risk of bias tool for animal studies. BMC Med Res Methodol.

[17] McGuinness LA, Higgins JP (2021). Visualization (robvis): an R package and Shiny web app for visualizing risk‐of‐bias assessments. Res Synth Methods.

[18] Zhao S, Zhang J, Zhu M, Zhang Y, Liu Z, Tao C (2015). Three-dimensional printed strontium-containing mesoporous bioactive glass scaffolds for repairing rat critical-sized calvarial defects. Acta Biomater.

[19] Balbinot GS, Leitune VCB, Ponzoni D, Collares FM (2019). Bone healing with niobium-containing bioactive glass composition in rat femur model: A micro-CT study. Dent Mater.

[20] Souza LPL, Lopes JH, Ferreira ,FV, Martin RA, Bertran CA, Camilli JA (2020). Evaluation of effectiveness of 45S5 bioglass doped with niobium for repairing critical-sized bone defect in in vitro and in vivo models. J Biomed Mater Res A.

[21] Deng Z, Chen J, Lin B, Li J, Wang H, Wang D (2020). A novel 3D printed bioactive scaffolds with enhanced osteogenic inspired by ancient Chinese medicine HYSA for bone repair. Exp Cell Res.

[22] Bai X, Liu W, Xu L, Ye Q, Zhou H, Berg C (2021). Sequential macrophage transition facilitates endogenous bone regeneration induced by Zn-doped porous microcrystalline bioactive glass. J Mater Chem B.

[23] Dai Q, Li Q, Gao H, Yao ,L, Lin Z, Li D (2021). 3D printing of Cu-doped bioactive glass composite scaffolds promotes bone regeneration through activating the HIF-1α and TNF-α pathway of hUVECs. Biomater Sci.

[24] Lappalainen OP, Karhula SS, Haapea M, Kauppinen S, Finnilä M, Saarakkal S (2016). Micro-CT Analysis of Bone Healing in Rabbit Calvarial Critical-Sized Defects with Solid Bioactive Glass; Tricalcium Phosphate Granules or Autogenous Bone. J Oral Maxillofac Res.

[25] Zhang J, Guan ,J, Zhang C, Wang H, Huang W, Guo ,S (2015). Bioactive borate glass promotes the repair of radius segmental bon,e defects by enhancing the osteogenic differentiation of BMSCs. Biomed Mater.

[26] Nommeots-Nomm A, Labbaf S, Devlin A, Todd N, Geng H, Solanki AK (2017). Highly degradable porous melt-derived bioactive glass foam scaffolds for bone regeneration. Acta Biomater.

[27] Knabe C, Mele A, Kann ,PH, Peleska ,B, Adel-Khattab D, Renz H (2017). Effect of sex-hormone levels; sex; body mass index and other host factors on human craniofacial bone regeneration with bioactive tricalcium phosphate grafts. Biomaterials.

[28] Gao H, Huang J, Wei Q, He C (2023). Advances in Animal Models for Studying Bone Fracture Healing. Bioengineering.

[29] Serdar CC, Cihan M, Yücel D, Serdar MA (2021). Sample size; power and effect size revisited: simplified and practical approaches in pre-clinical; clinical and laboratory studies. Biochem Med.

[30] Rizwan M, Hamdi M, Basirun W (2017). Bioglass® 45S5‐based composites for bone tissue engineering and functional applications. J Biomed Mater Res Part A.

[31] Banerjee A, Pluddemann A, O’Sullivan J, Nunan D. (2019). Catalogue Of Bias.

[32] Cardoso JR, Pereira LM, Iversen MD, Ramos AL. (2014). What is gold standard and what is ground truth?. Dental Press J Orthod.

